# Supplementary material for: Pleiotropic effect of the ABCG2 gene in gout: involvement in serum urate levels and progression from hyperuricemia to gout
Source: Arthritis Res Ther. 2020 Mar 12;22:45. doi: 10.1186/s13075-020-2136-z (PMC7069001; doi:10.1186/s13075-020-2136-z)
Supplement: Supplementary file 1 — Table S1. Characteristics of sample sets. (DOCX 20 kb) [file 13075_2020_2136_MOESM1_ESM.docx]

**Supplemental Table 1** Characteristics of sample sets

|  | European (N=16049) | | | Combined Polynesian (N=1608) | | |
| --- | --- | --- | --- | --- | --- | --- |
|  | Gout | HU | NU | Gout | HU | NU |
| N | 1699 | 2422 | 11928 | 912 | 202 | 494 |
| N Male (%) | 1439 (84.7) | 1909 (78.8) | 4773 (40.0) | 750 (82.2) | 147 (72.8) | 177 (35.8) |
| Age at recruitment, yrs – mean (sd) | 62.99 (12.9) | 54.79 (12.7) | 53.35 (12.9) | 53.22 (13.2) | 43.21 (15.4) | 44.86 (14.6) |
| Urate at recruitment, mmol/l - mean (sd) | 0.41 (0.13) *[85.2]** | 0.47 (0.05) | 0.3 (0.07) | 0.43 (0.12) *[90.6]* | 0.48 (0.05) | 0.33 (0.06) |
| Highest recorded urate, mmol/l - mean (sd)^ | 0.48 (0.14) *[95.5]* | 0.47 (0.05) | 0.3 (0.07) | 0.54 (0.12) *[95.3]* | 0.49 (0.06) | 0.34 (0.06) |
| BMI, kg/m^2^ - mean (sd) | 30.34 (7.09) *[87.5]* | 29.13 (4.93) *[80.6]* | 26.25 (4.72) *[81.3]* | 36.31 (8.07) *[94.4]* | 35.46 (7.34) *[98.0]* | 31.89 (7.09) *[96.6]* |
| Age of gout onset, yrs - mean (sd) | 47.6 (15.9) *[85.8]* | - | - | 38.80 (14.38) *[94.4]* | - | - |
| N attacks in past year - mean (sd) | 4.52 (8.59) *[54.9]* | - | - | 6.87 (10.75) *[92.1]* | - | - |
| N *rs2231142* genotypes available (%) | 1637 (96.4) | 2378 (98.2) | 11604 (97.3) | 912 (100) | 202 (100) | 494 (100) |
| N *rs10011796* genotypes available (%) | 1580 (93.0) | 2422 (100) | 11927 (100) | 910 (99.8) | 202 (100) | 494 (100) |
| N *rs11942223* genotypes available (%) | 1576 (92.8) | 2422 (100) | 11927 (100) | 912 (100) | 202 (100) | 494 (100) |

*All individuals had age and sex data at a minimum. All HU and NU controls had urate at recruitment. Some gout patients did not have urate at recruitment. Figures in italic square brackets represent the proportion of the sample set for which data were available.

^Highest recorded urate is the maximum urate value available in the data across variables pre-ULT urate, urate at diagnosis, urate at recruitment, urate in medical records. Individuals may have none (gout patients only), just one or all of these urate measures.
