# Supplementary material for: Pleiotropic effect of the ABCG2 gene in gout: involvement in serum urate levels and progression from hyperuricemia to gout
Source: Arthritis Res Ther. 2020 Mar 12;22:45. doi: 10.1186/s13075-020-2136-z (PMC7069001; doi:10.1186/s13075-020-2136-z)
Supplement: Supplementary file 2 — Table S2. Association analysis of rs2231142, rs10011796 (ABCG2) and rs11942223 (SLC2A9) in European and NZ Polynesian sample sets with the risk of gout in males only. (DOCX 29 kb) [file 13075_2020_2136_MOESM2_ESM.docx]

**Supplemental Table 2** Association analysis of *rs2231142, rs10011796 (ABCG2)* and *rs11942223* *(SLC2A9)* in European and NZ Polynesian sample sets with the risk of gout in males only

| **SNP** | **Raw N** | **Genotype, N (%)**^†^ | | | **Effect allele,**  **N (%)** | **Gout vs. HU*^**  **OR (95% CI), *P*** | **Gout vs. All Controls***  **OR (95% CI), *P*** | **HU vs. NU***  **OR, 95% CI, *P*** |
| --- | --- | --- | --- | --- | --- | --- | --- | --- |
| ***rs2231142*** |  | **GG** | **GT** | **TT** | **T** |  |  |  |
| European gout | 1388 | 843 (60.7) | 475 (34.2) | 70 (5.0) | 615 (22.2) | 1.91 (1.67-2.20), *4.7E-20*  1.96 (1.71-2.26), *4.8E-21* | 2.53 (2.26-2.84), *1.6E-58* | - |
| European HU | 1872 | 1398 (74.7) | 443 (23.7) | 31 (1.7) | 505 (13.5) |  |  | 1.60 (1.42-1.80), *7.3E-15* |
| European NU | 4642 | 3861 (83.2) | 739 (15.9) | 42 (0.9) | 823 (8.9) | - |  |  |
| WP gout | 330 | 83 (25.2) | 170 (51.5) | 77 (23.3) | 324 (49.1) | 1.95 (1.23- 3.17), *5.4E-03*  1.81 (1.10-3.03), *0.022* | 2.60 (1.81-3.81), *4.3E-07* | - |
| WP HU | 66 | 32 (48.5) | 24 (36.4) | 10 (15.2) | 44 (33.3) |  |  | 2.10 (1.15-4.01), *0.020* |
| WP NU | 68 | 40 (58.8) | 24 (35.3) | 4 (5.9) | 32 (23.5) | - |  |  |
| EP gout | 420 | 333 (79.3) | 86 (20.5) | 1 (0.2) | 88 (10.5) | 2.86 (1.28-7.41), *0.017*  3.03 (1.32-8.00) *0.015* | 3.56 (1.95-6.94), *7.8E-05* | - |
| EP HU | 81 | 74 (91.4) | 7 (8.6) | 0 (0.0) | 7 (4.3) |  |  | 1.38 (0.41- 4.64), *0.59* |
| EP NU | 109 | 102 (93.6) | 7 (6.4) | 0 (0.0) | 7 (3.2) | - |  |  |
| ***rs10011796*** |  | **CC** | **CT** | **TT** | **T** |  |  |  |
| European gout | 1335 | 278 (20.8) | 646 (48.4) | 411 (30.8) | 1468 (55.0) | 1.43 (1.28-1.58), *3.7E-06*  1.42 (1.28-1.59), *3.7E-11* | 1.59 (1.46-1.74), *1.1E-25* | - |
| European HU | 1909 | 525 (27.5) | 945 (49.5) | 439 (23.0) | 1823 (47.7) |  |  | 1.20 (1.11-1.29), *3.7E-06* |
| European NU | 4773 | 1511 (31.7) | 2381 (49.9) | 881 (18.5) | 4143 (43.4) | - | - |  |
| WP gout | 330 | 35 (10.61 | 145 (43.9) | 150 (45.5) | 445 (67.4) | 1.09 (0.70-1.69), *0.69*  1.03 (0.64-1.66), *0.89* | 1.18 (0.84-1.64), *0.34* | - |
| WP HU | 66 | 7 (10.6) | 32 (48.5) | 27 (40.9) | 86 (65.2) |  |  | 1.12 (0.63-1.99), *0.70* |
| WP NU | 68 | 8 (11.8) | 34 (50.0) | 26 (38.2) | 86 (63.2) | - |  |  |
| EP gout | 418 | 73 (17.5) | 204 (48.8) | 141 (33.7) | 486 (58.1) | 0.95 (0.65-1.38), *0.78*  0.89 (0.60-1.31), *0.55* | 0.94 (0.72-1.22), *0.63* | - |
| EP HU | 81 | 14 (17.3) | 34 (42.0) | 33 (40.7) | 100 (61.7) |  |  | 1.06 (0.69-1.63), *0.78* |
| EP NU | 109 | 19 (17.3) | 49 (45.0) | 41 (37.6) | 131 (60.1) | - |  |  |
| ***rs11942223*** |  | **TT** | **TC** | **CC** | **C** |  |  |  |
| European gout | 1332 | 1168 (74.1) | 382 (24.2) | 26 (1.6) | 373 (14.0) | 0.70 (0.60-0.81), *1.6E-06*  0.73 (0.61-0.82), *3.4E-06* | 0.56 (0.50-0.63), *9.9E-21* | - |
| European HU | 1909 | 1658 (68.5) | 701 (28.9) | 63 (2.6) | 694 (18.2) |  |  | 0.71 (0.65-0.79), *7.07E-12* |
| European NU | 4772 | 7101 (59.5) | 4220 (35.4) | 606 (5.1) | 2249 (23.6) | - |  |  |
| WP gout | 330 | 314 (95.2) | 16 (4.9) | 0 (0.0) | 16 (2.4) | 0.92 (0.22-6.31), *0.91*  0.88 (0.19-6.30), *0.88* | 0.95 (0.33-3.16), *0.93* | - |
| WP HU | 66 | 64 (97.0) | 2 (3.0) | 0 (0.0) | 2 (1.5) |  |  | 0.54 (0.06-3.71), *0.53* |
| WP NU | 68 | 65 (95.6) | 3 (4.4) | 0 (0.0) | 3 (2.2) | - |  |  |
| EP gout | 420 | 386 (91.9) | 31 (7.4) | 3 (0.7) | 37 (4.4) | 0.97 (0.40-2.78), *0.95*  1.10 (0.43-3.26), *0.86* | 1.18 (0.61-2.45), *0.63* | - |
| EP HU | 81 | 74 (91.4) | 7 (8.6) | 0 (0.0) | 7 (4.3) |  |  | 2.56 (0.75-8.92), *0.13* |
| EP NU | 109 | 102 (93.6) | 7 (6.4) | 0 (0.0) | 7 (3.2) | - |  |  |

*European sample set adjusted by age. Polynesian sample sets additionally adjusted by PCs 1-10.

^For Gout vs. HU analyses, the top figure is before adjustment by highest recorded serum urate, and the bottom figure is after adjustment.
